# Influences of Rhythm- and Timbre-Related Musical Features on Characteristics of Music-Induced Movement

**DOI:** 10.3389/fpsyg.2013.00183

**Published:** 2013-04-12

**Authors:** Birgitta Burger, Marc R. Thompson, Geoff Luck, Suvi Saarikallio, Petri Toiviainen

**Affiliations:** ^1^Department of Music, Finnish Centre of Excellence in Interdisciplinary Music Research, University of JyväskyläJyväskylä, Finland

**Keywords:** music-induced movement, dance, motion capture, musical feature extraction, pulse clarity, spectral flux

## Abstract

Music makes us move. Several factors can affect the characteristics of such movements, including individual factors or musical features. For this study, we investigated the effect of rhythm- and timbre-related musical features as well as tempo on movement characteristics. Sixty participants were presented with 30 musical stimuli representing different styles of popular music, and instructed to move along with the music. Optical motion capture was used to record participants’ movements. Subsequently, eight movement features and four rhythm- and timbre-related musical features were computationally extracted from the data, while the tempo was assessed in a perceptual experiment. A subsequent correlational analysis revealed that, for instance, clear pulses seemed to be embodied with the whole body, i.e., by using various movement types of different body parts, whereas spectral flux and percussiveness were found to be more distinctly related to certain body parts, such as head and hand movement. A series of ANOVAs with the stimuli being divided into three groups of five stimuli each based on the tempo revealed no significant differences between the groups, suggesting that the tempo of our stimuli set failed to have an effect on the movement features. In general, the results can be linked to the framework of embodied music cognition, as they show that body movements are used to reflect, imitate, and predict musical characteristics.

## Introduction

Music makes us move. While listening to music, we often move our bodies in a spontaneous fashion. Keller and Rieger (2009), for example, stated that simply listening to music can induce movement, and in a self-report study conducted by Lesaffre et al. (2008), most participants reported moving when listening to music. Janata et al. (2011) reported a study in which they asked participants to tap to the music and found that participants not only moved the finger/hand, but also other body parts, such as feet and head. Additionally, the tapping condition (isochronous versus free tapping) influenced the amount of movement: the more “natural” the tapping condition, the more movement was exhibited.

In general, people tend to move to music in an organized way by, for example, mimicking instrumentalists’ gestures, or rhythmically synchronizing with the pulse of the music by tapping their foot, nodding their head, or moving their whole body in various manners (Godøy et al., 2006; Leman and Godøy, 2010). Moreover, Leman (2007, p. 96) suggests, “Spontaneous movements [to music] may be closely related to predictions of local bursts of energy in the musical audio stream, in particular to the beat and the rhythm patterns.” Such utilization of the body is the core concept of embodied cognition, which claims that the body is involved in or even required for cognitive processes (e.g., Lakoff and Johnson, 1980, 1999, or Varela et al., 1991). Human cognition is thus highly influenced by the interaction between mind/brain, sensorimotor capabilities, body, and environment. Following this, we can approach music (or musical involvement) by linking our perception of it to our body movement (Leman, 2007). One could postulate that our bodily movements reflect, imitate, help to parse, or support understanding the structure and content of music. Leman suggests that corporeal articulations could be influenced by three (co-existing) components or concepts: “Synchronization,” “Embodied Attuning,” and “Empathy,” which differ in the degree of musical involvement and in the kind of action-perception couplings employed. “Synchronization” forms the fundamental component, as synchronizing to a beat is easy and spontaneous. The beat serves as the basic musical element, from which more complex structures emerge. Leman furthermore suggests the term “inductive resonance,” referring to the use of movements for active control, imitation, and prediction of beat-related features in the music (the opposite of passively tapping to a beat) as the first step in engaging with the music. The second component, “Embodied Attuning,” concerns the linkage of body movement to musical features more complex than the basic beat, such as melody, harmony, rhythm, tonality, or timbre. Following this idea, movement could be used to reflect, imitate, and navigate within the musical structure in order to understand it. Finally, “Empathy” is seen as the component that links musical features to expressivity and emotions. In other words, the listener feels and identifies with the emotions expressed in the music and imitates and reflects them by using body movement.

It has been argued that music and dance have evolved together in most cultures (Arom, 1991; Cross, 2001) and are crucial elements of most social and collective human behavior (Brown et al., 2000). Furthermore, most cultures have developed coordinated dance movements to rhythmically predictable music (Nettl, 2000). There is neurobiological evidence for a connection between rhythmic components of music and movement (e.g., Grahn and Brett, 2007; Bengtsson et al., 2009; Chen et al., 2009; Grahn and Rowe, 2009), which has led to the assumption that humans prefer music that facilitates synchronization and respond to it with movement (Madison et al., 2011). Phillips-Silver and Trainor (2008) showed in their study that especially head movements were found to bias metrical encoding of rhythm and meter perception.

The increasing opportunities of quantitative research methods for recording and analyzing body movement have offered new insights and perspectives for studying such movements. A number of studies have investigated (professional) dance movements using quantitative methods. Camurri et al. (2003, 2004), for instance, developed a video analysis tool to recognize and classify expressive and emotional gestures in professional dance performances. Jensenius (2006) developed the technique of “motiongrams” for visualizing and analyzing movement and gestures. Stevens et al. (2009) studied movements of professional dancers regarding time keeping with and without music using optical motion capture recordings. Optical motion capture was also employed by Naveda and Leman (2010) and Leman and Naveda (2010) who investigated movement in samba and Charleston dancing, focusing on spatiotemporal representations of dance gestures as movement trajectories.

Besides professional dance, several studies were dedicated to more general tasks and behaviors involving music-induced movement, such as movement of infants or laymen dancers. Zentner and Eerola (2010) investigated infants’ ability to bodily synchronize with musical stimuli, finding that infants showed more rhythmic movement to music and metrical stimuli than to speech suggesting a predisposition for rhythmic movement to music and other metrical regular sounds. Eerola et al. (2006) studied toddlers’ corporeal synchronization to music, finding three main periodic movement types being at times synchronized with pulse of the music. Toiviainen et al. (2010) investigated how music-induced movement exhibited pulsations on different metrical levels, and showed that eigenmovements of different body parts were synchronized with different metric levels of the stimulus. Luck et al. (2010) studied the influence of individual factors such as personality traits and preference on musically induced movements of laypersons’ dancing, finding several relationships between personality traits and movement characteristics. Van Dyck et al. (2010) found that an increased presence of the bass drum tends to increase listeners’ spontaneous movements. However, systematic investigations targeting the relationships between musical features, particularly rhythm-related features, and human movement characteristics have not been conducted. Such an investigation would reveal additional information as to how music shapes movement. Additionally, finding dependencies of musical structure and body movements that are consistent between individuals would support the notion of embodied music cognition (Leman, 2007).

Rhythmic music is based on beats, which can be physically characterized as distinct energy bursts in time. If such beats occur as regular and repetitive temporal patterns, they give rise to a percept of pulse. Beat and pulse structures can be regarded as the basic metrical structure in music from which more complex temporal structures, such as rhythm, emerge. This is typically achieved by subdividing the basic metrical structure in smaller and larger units of varying lengths, constructing a metrically interlocked grid with events on different temporal levels (Parncutt, 1994). These rhythmic structures can vary, for example, in the degree of pulse clarity. Pulse clarity estimates, on a large time scale, how clearly the underlying pulsation in music is perceivable and can therefore be regarded as a measure for the underlying periodicity of the music (Lartillot et al., 2008). Another aspect of rhythmic structure is covered by spectro-temporal features, such as the sub-band spectral flux, which has been found to be among the most important features contributing to polyphonic timbre perception (Alluri and Toiviainen, 2010). Spectral flux measures spectral change, which, when taken separately for different sub-bands, is related to certain elements of the rhythm (i.e., rhythmic elements created by instruments within the frequency range of the respective sub-band). It could be that sub-band flux is a crucial feature not only in a (passive) listening situation, but also in a (active) movement situation. Furthermore, other timbral characteristics, such as percussiveness, could have an influence on movement responses to music. For instance, high amounts of percussive elements in music could result in fast movements, reflecting the way such sounds are often produced. Following these notions, it could be assumed that variations in such musical features not only increase or decrease the amount of movement, but also change the kinds and properties of the movements. In line with the embodied music cognition approach (Leman, 2007), the movements could reflect and imitate the rhythmical and timbral structure of the music.

Besides features such as pulse clarity, tempo is an important factor contributing to the perception of rhythm (Fraisse, 1982). Tempo is the speed at which beats are repeated, the underlying periodicity of music. Tempo is usually measured in beats per minute (bpm). A large body of research has been conducted on listeners’ abilities to perceive different tempi, synchronize to them, and reproduce them in tapping tasks (for a review see Repp, 2005). Free tapping tasks have found that a majority of participants tapped at a rate close to 600 ms, though the individual rates differed considerably (Fraisse, 1982). Synchronizing to steady, periodic beat stimuli is possible at a wide range of tempi, however it is most regular and accurate for intervals around 400–500 ms (Collyer et al., 1992), respectively 400–800 ms (Fraisse, 1982), while with slower and faster tempo the time between two taps becomes more variable. Moelants (2002) suggested 120 bpm as the preferred tempo – the tempo where tempo perception is considered to be optimal and appears most natural. Interesting to note here is that literature often draws links between spontaneous/preferred tempo and repeated motor activities, such as walking, for which the spontaneous duration of steps is around 500–550 ms (Murray et al., 1964; Fraisse, 1982; MacDougall and Moore, 2005). Walking has been suggested as “a fundamental element of human motor activity” (Fraisse, 1982, p. 152) and could therefore serve as an origin of preferred tempo perception. Following these considerations, it could furthermore be assumed that music with tempi around 110–120 bpm stimulate movement more than music with other tempi.

In order to investigate relationships between rhythmic and timbral aspects of music and movements that such music induces, we conducted a motion capture experiment, in which participants were asked to move to music that differed in tempo and in rhythm- and timbre-related features, such as pulse clarity, percussiveness, and spectral flux of low and high frequency ranges. We were interested in two main aspects: first, whether movement and musical characteristics are somehow related, and more particular, which body parts and movement types reflect different musical characteristics. Second, whether the tempo of music has an influence on the movement. Following the notion of embodied (music) cognition we assumed the movement to reflect aspects of the music. First, we expected movements to indicate the beat structure, in particular that a clear beat would be embodied by increased speed of movements. Second, we anticipated that hand and arm movements, having the most freedom when moving/dancing, would play an important role in reflecting musical characteristics. Finally, we assumed movement features to resemble the preferred tempo insofar, as tempi around 120 bpm might encourage participants to move differently than tempi slower or faster than 120 bpm.

## Materials and Methods

### Participants

A total of 64 participants took part in the study. Four participants were excluded from further analysis due to incomplete data. Thus, 60 participants remained for subsequent analyses (43 female, 17 male, average age: 24, SD of age: 3.3). Six participants had received formal music education, while four participants had a formal background in dance tuition. Participation was rewarded with a movie ticket. All participants gave their informed consent prior to their inclusion in the study and they were free to discontinue the experiment at any point. Ethical permission for this study was not needed, which was according to the guidelines stated by the university ethical board.

### Stimuli

Participants were presented with 30 randomly ordered musical stimuli representing the following popular music genres: techno, Pop, Rock, Latin, Funk, and Jazz. An overview of the stimuli can be found in the Appendix. All stimuli were 30 s long, non-vocal, and in 4/4 time, but differed in their rhythmic complexity, pulse clarity, mode, and tempo. The stimulus length was chosen to keep the experiment sufficiently short while having stimuli that were long enough to induce movement.

### Apparatus

**Figure 1:**
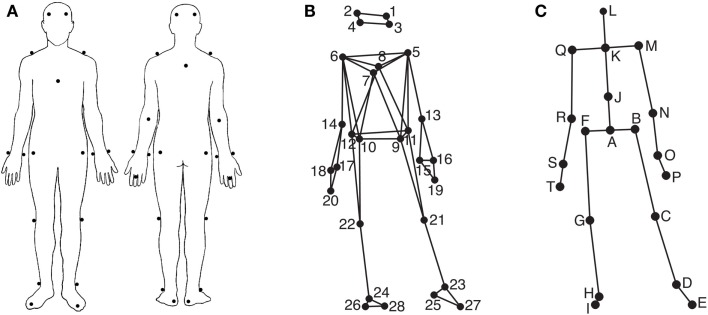
**Marker and joint locations**. **(A)** Anterior and posterior view of the marker placement on the participants’ bodies; **(B)** Anterior view of the marker locations as stick figure illustration; **(C)** Anterior view of the locations of the secondary markers/joints used in the analysis.

Participants’ movements were recorded using an eight-camera optical motion capture system (Qualisys ProReflex), tracking, at a frame rate of 120 Hz, the three-dimensional positions of 28 reflective markers attached to each participant. The locations of the markers can be seen in Figures 1A,B. The location of the markers were as follows (L, left; R, right; F, front; B, back): 1, LF head; 2, RF head; 3, LB head; 4, RB head; 5, L shoulder; 6, R shoulder; 7, sternum; 8, spine (T5); 9, LF hip; 10, RF hip; 11, LB hip; 12, RB hip; 13, L elbow; 14, R elbow; 15, L wrist/radius; 16, L wrist/ulna; 17, R wrist/radius; 18, R wrist/ulna; 19, L middle finger; 20, R middle finger; 21, L knee; 22, R knee; 23, L ankle; 24, R ankle; 25, L heel; 26, R heel; 27, L big toe; 28, R big toe. The musical stimuli were played back via a pair of Genelec 8030A loudspeakers using a Max/MSP patch running on an Apple computer. The room sound was recorded with two overhead microphones positioned at a height of approximately 2.5 m. This microphone input, the direct audio signal of the playback, and the synchronization pulse transmitted by the Qualisys cameras when recording, were recorded using ProTools software in order to synchronize the motion capture data with the musical stimulus afterward. Additionally, four Sony video cameras were used to record the sessions for reference purposes.

### Procedure

Participants were recorded individually and were asked to move to the presented stimuli in a way that felt natural. Additionally, they were encouraged to dance if they wanted to, but were requested to remain in the center of the capture space indicated by a 115 × 200 cm carpet.

### Movement feature extraction

In order to extract various kinematic features, the MATLAB Motion Capture (MoCap) Toolbox (Toiviainen and Burger, 2011) was used to first trim the data to the duration of each stimulus and, following this, derive a set of 20 secondary markers – subsequently referred to as joints – from the original 28 markers. The locations of these 20 joints are depicted in Figure 1C. The locations of joints C, D, E, G, H, I, M, N, P, Q, R, and T are identical to the locations of one of the original markers, while the locations of the remaining joints were obtained by averaging the locations of two or more markers; joint A, midpoint of the four hip markers (called root in the further analysis); B, midpoint of markers 9 and 11 (left hip); F, midpoint of markers 10 and 12 (right hip); J, midpoint of breastbone, spine, and the hip markers (midtorso); K, midpoint of shoulder markers (manubrium), L, midpoint of the four head markers (head); O, midpoint of the two left wrist markers (left wrist); S, midpoint of the two right wrist markers (right wrist). Thereafter, eight movement variables were extracted from the joint location data to cover different movement characteristics, body parts, and movement types.

– Speed of Center of Mass, Head, both Hands, and both Feet: for calculating the speed of the movement, we applied numerical differentiation based on the Savitzky and Golay (1964) smoothing FIR filter with a window length of seven samples and a polynomial order of two. These values were found to provide an optimal combination of precision and smoothness in the time derivatives. For the Speed of Head, Hands, and Feet, the data were transformed into a local coordinate system, in which joint A was located at the origin, and segment BF had zero azimuth.
– Hand Distance: this feature relates to the distance between both hands.
– Amount of Movement: this feature is based on the traveled distance of all markers and gives a measure for the total amount of movement (data were also transformed into the local coordinate system).
– Hip Wiggle: this feature is defined as the mean absolute angular velocity of the hips around the anteroposterior axis.
– Shoulder Wiggle: this feature is defined as the mean absolute angular velocity of the shoulder around the anteroposterior axis.

Subsequently, the instantaneous values of each variable were averaged for each stimulus presentation. This yielded a total of eight statistical movement features for each of the 60 participants and 30 stimuli.

### Musical feature extraction

To investigate the effect of rhythm- and timbre-related features on music-induced movement, we performed computational feature extraction analysis of the stimuli used in the experiment. To this end, four musical features were extracted from the stimuli using the MATLAB MIRToolbox (version 1.4) (Lartillot and Toiviainen, 2007).

**Figure 2:**
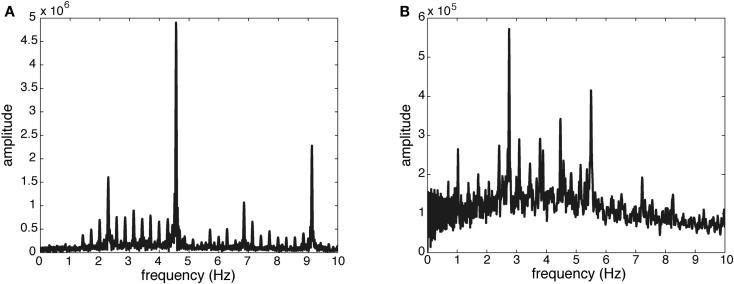
**Fluctuation spectra of two stimuli used in the study**. **(A)** Peaks at a regular distance of 0.28 Hz, with the highest peak at 4.56 Hz and other clear peaks at 2.29, 6.85, and 9.13 Hz, suggesting clear pulses and periodicity (stimulus 1, see Appendix). **(B)** Markedly lower magnitude values, a less periodic pattern of peaks, and more noise, suggesting low pulse clarity (stimulus 21, see Appendix).

**Figure 3:**
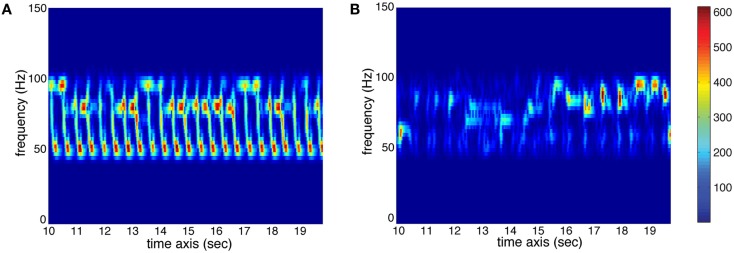
**Spectrograms (sec. 10–20) of sub-band no. 2 (50–100 Hz) of two stimuli used in the study**. **(A)** High amount of temporal change (red represents high energy at the respective time and frequency, whereas blue represents low energy; see color bar) resulting in high value for Sub-Band Flux (stimulus 26, see Appendix). **(B)** Low amount of temporal change resulting in low Sub-Band Flux (stimulus 5, see Appendix).

– Pulse Clarity: this feature indicates the strength of rhythmic periodicities and pulses in the signal, estimated by the relative Shannon entropy of the fluctuation spectrum (Pampalk et al., 2002). In this context, entropy can be understood as a measure of the degree of peakiness of the spectrum. Music with easily perceived beat has a distinct and regular fluctuation spectrum, which has low entropy. Thus, high pulse clarity is associated with low fluctuation entropy. To illustrate this measure of Pulse Clarity, the fluctuation spectra for high and low Pulse Clarity are shown in Figure 2.
– Sub-Band Flux: this feature indicates to which extend the spectrum changes over time. For the calculation, the stimulus is divided into 10 frequency bands, each band containing one octave in the range of 0–22050 Hz. The Sub-Band Flux is then calculated for each of these ten bands separately by calculating the Euclidean distances of the spectra for each two consecutive frames of the signal (Alluri and Toiviainen, 2010), using a frame length of 25 ms and an overlap of 50% between successive frames and then averaging the resulting time-series of flux values. For the current analysis, we used two frequency bands: band no. 2 (50–100 Hz) and band no. 9 (6400–12800 Hz). High flux in the low frequency bands is produced by instruments such as kick drum and bass guitar, whereas high flux in the high frequency bands is produced by instruments such as cymbals or hihats. Two spectrograms of sub-band no. 2 are displayed in Figure 3 to show the difference between high and low amounts of Sub-Band Flux.
– Percussiveness: for this feature, the slopes of the amplitude envelopes at note onsets are estimated and then averaged across all slopes of the signal. The steeper the slope, the more percussive and accentuated the sound.

Additionally, the Tempo of the stimuli was assessed in a separate tapping experiment, in which ten participants tapped to the 30 stimuli. The average tempo was evaluated by taking the median value of all intertap intervals.

## Results

In order to perform further analysis, we first checked for consistency between participants by calculating Intraclass Correlations (cf., Shrout and Fleiss, 1979) for each movement feature separately. Intraclass correlations operate on data that are structured in groups (as opposed to standard correlation procedures that assume data to be structured as paired observations) and indicate how strongly units of the same group (in this case the values of one movement feature for all participants and songs) resemble each other. The values are presented in Table 1.

**Table 1:** **Results of the intraclass correlations for each of the movement features**.

Movement feature	*r*
Speed of center of mass	0.90***
Speed of head	0.89***
Speed of hands	0.91***
Speed of feet	0.90***
Hand distance	0.62***
Amount of movement	0.94***
Hip wiggle	0.95***
Shoulder wiggle	0.88***

***p < 0.001.

All intraclass correlations resulted in highly significant coefficients. Therefore, we concluded that participants’ movements for each song were similar enough to justify averaging the movement features across participants, yielding one value for each stimulus presentation.

Subsequently, we correlated the movement features with Pulse Clarity, Low and High Frequency Spectral Flux, and Percussiveness. Due to the relatively large number of correlations (32), we applied Bonferroni correction to avoid false positive errors in case of multiple comparisons. The results are displayed in Table 2.

**Table 2:** **Results of the correlation between movement and musical features**.

	Pulse clarity	Spectral flux SB 2	Spectral flux SB 9	Percussiveness
Speed of center of mass	0.67**	0.51	0.52	0.56*
Speed of head	0.52	0.73***	0.64**	0.67**
Speed of hands	0.54	0.46	0.65**	0.57*
Speed of feet	0.55*	0.31	0.46	0.45
Hand distance	0.34	0.29	0.57*	0.55*
Amount of movement	0.62**	0.44	0.57*	0.56*
Hip wiggle	0.58*	0.25	0.47	0.46
Shoulder wiggle	0.67**	0.39	0.48	0.61*

*p < 0.05, **p < 0.01, ***p < 0.001.

As can be seen, Pulse Clarity shows significant positive correlations with Speed of Center of Mass [*r*(30) = 0.67, *p* < 0.01], Speed of Feet [*r*(30) = 0.55, *p* < 0.05], Amount of Movement [*r*(30) = 0.62, *p* < 0.01], Hip Wiggle [*r*(30) = 0.58, *p* < 0.05], and Shoulder Wiggle [*r*(30) = 0.67, *p* < 0.01]. Thus, for music with a clear pulse, participants tended to move the center of the body and feet faster, wiggled more with hips and shoulders, and used an increased amount of movement related to the whole body. For an illustration of these features, an animation of movements performed by one participant for the stimulus with the highest value for Pulse Clarity (stimulus 1, see Appendix) can be found in the “PulseClarity.mov” in Supplementary Material. The selected participant exhibited high values for all the movement features that were shown to be characteristic for high Pulse Clarity.

Spectral Flux of Sub-band no. 2 exhibited a high positive correlation with Speed of Head [*r*(30) = 0.73, *p* < 0.001], suggesting that stimuli with strong spectral flux in the low frequencies are related to fast head movements. For illustration, an animation of movements performed by one participant for the stimulus with the highest value for Spectral Flux of Sub-band no. 2 (stimulus 26, see Appendix) can be found in the “SubBandFluxNo2.mov” in Supplementary Material.

Spectral Flux of Sub-band no. 9 showed significant positive correlations with Speed of Head [*r*(30) = 0.64, *p* < 0.01], Speed of Hands [*r*(30) = 0.65, *p* < 0.01], Hand Distance [*r*(30) = 0.57, *p* < 0.05], and Amount of Movement [*r*(30) = 0.57, *p* < 0.05], indicating that, for stimuli with strong spectral flux in the high frequencies, participants moved their head and hands faster, had a larger distance between the hands, and used an increased amount of movement. To illustrate these features, an animation of movements performed by one participant for the stimulus with the highest value for Spectral Flux of Sub-band no. 9 (stimulus 18, see Appendix) can be found in the “SubBandFluxNo9.mov” in Supplementary Material.

Percussiveness exhibited positive correlations with Speed of Center of Mass [*r*(30) = 0.56, *p* < 0.05], Speed of Head [*r*(30) = 0.67, *p* < 0.01], Speed of Hands [*r*(30) = 0.57, *p* < 0.05], Hand Distance [*r*(30) = 0.55, *p* < 0.05], Amount of Movement [*r*(30) = 0.56, *p* < 0.05], and Shoulder Wiggle [*r*(30) = 0.61, *p* < 0.01]. Thus, for stimuli containing a high amount of percussive elements, participants moved their center of mass, head, and hands faster, had a larger distance between their hands, used an increased amount of movement, and wiggled more with their shoulders. For an illustration of these features, an animation of movements performed by one participant for the stimulus with the highest value for Percussiveness (stimulus 3, see Appendix) can be found in the “Percussiveness.mov” in Supplementary Material.

As we assumed a non-linear relationship between Tempo and the movement features, we excluded Tempo from the correlational analysis. Instead, we rank-ordered the stimuli based on their tempi obtained from the tapping experiment and picked the five slowest, the five medial, and the five fastest stimuli to perform a series of ANOVAs on these three groups. The tempi of the stimuli in these groups ranged from 73 to 87 bpm, from 113 to 125 bpm, and from 138 to 154 bpm. The results of the ANOVAs are shown in Table 3.

**Table 3:** **Results from the series of ANOVAs, testing for differences in the movement features between the three tempo groups**.

	*F*(2, 897)	*p*
Speed of center of mass	0.36	0.70
Speed of head	0.55	0.58
Speed of hands	0.58	0.56
Speed of feet	0.40	0.67
Hand distance	0.06	0.94
Amount of movement	1.35	0.26
Hip wiggle	1.64	0.19
Shoulder wiggle	0.42	0.66

As none of the ANOVAs showed significant differences between the groups, the tempo of our set of stimuli failed to have any significant effect on the movement features.

## Discussion

We investigated music-induced movement, focusing on the relationship between rhythm- and timbre-related musical features, such as Pulse Clarity and Spectral Flux, and movement characteristics of different body parts.

Pulse Clarity seemed to be embodied by using various movement types of different body parts, such as Speed of Center of Mass and Feet, Amount of Movement, and Hip and Shoulder Wiggle. Participants used an increasing amount of different movements and movement types of the whole body when the music contained easily and clearly perceivable pulses. Pulse Clarity might, therefore, influence body movement on a somewhat global, whole body level.

Low frequency (50–100 Hz) Spectral Flux was positively correlated to the Speed of Head. We observed that a high amount of low frequency Spectral Flux was associated with the presence of kick drum and bass guitar, creating strong low frequency rhythmic elements that are usually related to the beat period. A reason for the head being involved could be that, based on biomechanical properties, the head might be most prone to move to the beat level (cf. nodding to the beat or “head banging” in certain musical genres). Interesting to note here is that we found only one significant correlation, so it could be concluded that spectral changes in the low frequency ranges influences body movement on a rather local, specific level.

Spectral Flux in the high frequencies (6400–12800 Hz) was found to relate to Speed of Head and Hands, Hand Distance, and Amount of Movement. We observed that high frequency Spectral Flux was mainly influenced by the presence of hi-hat and cymbal sounds, creating the high frequency rhythmical figures and hence the “liveliness” of the rhythm. That could explain the dominance of hand-related movement characteristics, as the hands have the biggest movement freedom of the whole body and can therefore reflect fast rhythmic structures best.

Percussiveness correlated with Speed of Center of Mass, Head, and Hands, Hand Distance, Amount of Movement, and Shoulder Wiggle, suggesting that percussive elements in the music were related to movement of the upper body, especially to the hands as the most flexible body parts. Together with the findings for high frequency Spectral Flux, these results could indicate that timbral features not only affect movement responses to music, but more particular that the embodiment of these features is to a large proportion related to upper body and hand movement. Furthermore, high amounts of percussive elements seemed to be embodied with fast movement, supporting the assumption that the movement reflects the way such sounds are often produced.

The Tempo of the music failed to show any relationship with the movement features and could therefore not confirm our hypothesis that music around 120 bpm would stimulate movement differently than faster or slower music. However, this might be due to our selection of the stimuli. The variety of musical styles that was covered by the stimuli might have undermined the influence of the tempo. This issue thus remains an open question that requires further investigation.

The results can be linked to the framework of embodied music cognition and could provide support for Leman’s (2007) concepts of Synchronization, Inductive Resonance, and Embodied Attuning. The concepts of Synchronization and Inductive Resonance can be related to our findings regarding Pulse Clarity: clearly perceivable and strong beats might have a resonating effect on the overall body/torso movement and amount of movement, reflecting and imitating the clear beat structure, and making participants synchronize to the pulse. With less clear pulses, people might be less affected by the resonating effect, and are thus less encouraged to move. Concerning the correlations with Pulse Clarity, it is interesting to note that Speed of Feet correlated significantly with this feature, but not with any other musical feature. A possible explanation for this connection could be that foot movement is usually related to walking, with steps at a regular pace. In a musical context, the pulse of the music is related to a regular pace, so it could be argued that a clear and regular pulse of the music was embodied with using more extensive and synchronized foot movement, that is, stepping to the pulse (see Styns et al. (2007) for further connections between walking and beat patterns). However, our movement features do not reveal information about the actual synchronization to the pulse, and thus further analysis has to be performed to justify this interpretation. The torso movement (Center of Mass Speed, Hip Wiggle, Shoulder Wiggle) could also be part of the stepping-/walking-type movement of legs/feet, which would explain the significant correlations with Pulse Clarity. Furthermore, the feet are required to provide stability and an upright position, so they cannot move as free as, for instance, the hands. Thus, a connection of these body parts to the fundamental components of musical engagement, such as synchronization and resonance, makes sense, whereas the upper body parts (e.g., hands and head) could be expected to be more related to timbral and rhythmical structures of music (cf., Embodied Attuning, see next paragraph).

Moreover, the results could serve as an example for the concept of Embodied Attuning – movement-based navigation through the musical/rhythmic structures of the stimuli created by Sub-Band Flux and Percussiveness. It could be suggested that participants attune to strong spectral changes in low and high frequency ranges and to percussive elements with mainly head and hand movements, as these musical characteristics (related to rhythm) might be reflected and imitated best by using the upper extremities of the body (hand, head, and shoulder movement). Especially the hands (together with arms and shoulders) could be used to navigate through the music and to parse and understand the rhythmic/musical structure better.

Besides having the biggest freedom in movement as mentioned previously, the relation between hand/arm-related movement and several musical features might also occur due to knowledge and imagination of playing a musical instrument (Leman, 2007). One could postulate that participants have applied their own experience in playing an instrument to convert this knowledge into spontaneous hand and arm movement; in other words, such movements could have been used as instrumental gestures to reflect and imitate certain musical features (Leman, 2007). Both high frequency Sub-band Flux and Percussiveness were found to be related to hand movements. Such movement characteristics could reflect the way these sound qualities are produced. For instance, participants could have imagined playing the drums during moving to the music (cf., “Air Instruments,” Godøy et al., 2006). A follow-up study including an appropriate background questionnaire (covering use and experience of musical instruments) should be conducted to investigate this relationship in more detail.

Furthermore, the link between head movement and rhythm-related musical features might be based on the tendency to use head movements to spontaneously follow the rhythm of the music. This could be seen as another example of the concept of embodied attuning (Leman, 2007). An additional interpretation of such head movements could be related to the results by Phillips-Silver and Trainor (2008), who found head movement to bias meter perception, as well as by Trainor et al. (2009), who discovered the vestibular system to play a primal role in rhythm perception: movements of the head could therefore support rhythm perception and understanding.

Movements are used not only in musical contexts, but also in speech. Hand and head gestures are widely utilized to accompany speech and convey additional information. Hadar (1989) noted that such gestures are used, for example, to clarify or emphasize messages. Consequently, our participants could have used their hand and head movements in a similar fashion, i.e., to emphasize and elaborate the musical (e.g., rhythmic) structure or certain musical events.

We aimed at designing an ecological study, as far as this is possible with an optical motion capture system and a laboratory situation. To this end, we chose real music stimuli (pre-existing pop songs), accepting the downside that they were less controlled, very diverse, and more difficult to analyze, as computational analysis of complex musical stimuli is not yet as sufficiently developed as for simpler, i.e., monophonic, stimuli. Furthermore, the stimuli might contain relevant musical features that we missed to extract. However, this approach made it possible to present the participants with music that they were potentially familiar with, and that is played in dance clubs. One could assume that this kind of music would make them move more and in a more natural fashion than more artificial stimuli.

The movement characteristics chosen in this study cover only a small part of the possible movement characteristics and types. There are certainly stereotypical genre- and style-dependent movements that are rather culturally developed than intrinsic to the music. Examples of these kinds of movements would be head banging in rock music or swaying hips to Latin music. To get more insight into such movement patterns, gesture-based computational analysis of the movement could be performed in the future.

As Desmond (1993) noted, dance is related to cultural identity. Since the musical styles used in this study can all be characterized as popular music in the cultural region in which the data collection took place, different movement characteristics might be found with participants that are not as familiar with such musical styles as our participants were. Comparative studies would give insights into cultural differences and commonalities of music-induced movement characteristics.

The present study revealed relationships between rhythm- and timbre-related musical features and movement characteristics. In the future, we will investigate periodicity and synchronization of music-induced movement, as well as further relationships of movement characteristics and musical features, such as tonality features, as they play an important role for the perception of musical emotions. Additionally, the results obtained in this study regarding tempo call for further investigation. A new set of stimuli could be created to control for tempo and related styles/genres to investigate the relationship of tempo, musical style, and resulting movement features.

## Conflict of Interest Statement

The authors declare that the research was conducted in the absence of any commercial or financial relationships that could be construed as a potential conflict of interest.

## Supplementary Material

The Supplementary Material for this article can be found online at http://www.frontiersin.org/Auditory_Cognitive_Neuroscience/10.3389/fpsyg.2013.00183/abstract

## Appendix

### Song list

1. Alice Deejay: better Off Alone (Who Needs Guitars Anyway?) 2:40–2:54 (loop).
2. Andre Visior: speed Up 1:15–1:45.
3. Antibalas: who is this America Dem Speak of Today? (Who Is This America?) 1:00–1:29.
4. Arturo Sandoval: a Mis Abuelos (Danzon) 1:53–2:21.
5. Baden Powell: deixa (Personalidade) 1:11–1:41.
6. Brad Mehldau: wave/Mother Nature’s Son (Largo) 0:00–0:29.
7. Clifford Brown & Max Roach: the Blues walk (Verve Jazz Masters, Vol. 44: Clifford Brown & Max Roach) 2:01–2:31.
8. Conjunto Imagen: medley-Esencia de Guaguanco/Sonero (Ayer, Hoy y Manana) 2:18–2:48.
9. Dave Hillyard & The Rocksteady 7: Hillyard Street (Playtime) 0:15–0:45.
10. Dave Weckl: mercy, Mercy, Mercy (Burning for Buddy) 0:10–0:40.
11. Dave Weckl: tower of Inspiration (Master Plan) 0:00–0:30.
12. DJ Shadow: napalm Brain/Scatter Brain (Endtroducing …) 3:29–3:58.
13. Gangster Politics: gangster Politics (Guns & Chicks) 1:00–1:29.
14. Gigi D’Agostino: blablabla (L’Amour Toujours) 0:00–0:30.
15. Herbie Hancock: watermelon man (Cantaloupe Island) 0:00–0.30.
16. Horace Silver: the Natives Are Restless 0:00–0:29.
17. In Flames: scream (Come Clarity) 0:00–0:30.
18. Jean Roch: can You Feel it (Club Sounds Vol. 35) 0:33–1:01.
19. Johanna Kurkela: Hetki hiljaa (Hetki hiljaa) 3:22–3:52.
20. Juana Molina: tres cosas (Tres Cosas) 0:00–0:30.
21. Kings of Leon: closer (Only by the Night) 3:17–3:47.
22. Lenny Kravitz: live (5) 3:02–3:30.
23. Martha & The Vandellas: heat Wave (Heat Wave) 1:40–2:10.
24. Maynard Ferguson: fireshaker (Live From San Francisco) 0:00–0:28.
25. MIA: 20 Dollar (Kala) 0:17–0:45.
26. Nick Beat: techno Disco 2:26–2:56.
27. Panjabi MC: mundian To Bach Ke (Legalized) 0:47–1:06 (loop).
28. Patrick Watson: Beijing (Wooden Arms) 2:30–2:59.
29. The Rippingtons: weekend in Monaco (Weekend in Monaco) 1:13–1:42.
30. Yuri Buenaventura: salsa (Salsa Movie Soundtrack) 2:17–2:45.

## References

[B1] Alluri V. Toiviainen P. (2010). Exploring perceptual and acoustical correlates of polyphonic timbre. Music Percept. 27, 223–242 10.1525/mp.2010.27.3.223

[B2] Arom S. (1991). African Polyphony and Polyrhythm: Musical Structure and Methodology. Cambridge: Cambridge University Press

[B3] Bengtsson S. L. Ullén F. Ehrsson H. H. Hashimoto T. Kito T. Naito E. (2009). Listening to rhythms activates motor and premotor cortices. Cortex 45, 62–71 10.1016/j.cortex.2008.07.002 19041965

[B4] Brown S. Merker B. Wallin N. L. (2000). “An introduction to evolutionary musicology,” in The Origins of Music, eds Wallin N. L. Merker B. Brown S. (Cambridge, MA: MIT Press), 3–24

[B5] Camurri A. Lagerlöf I. Volpe G. (2003). Recognizing emotion from dance movement: comparison of spectator recognition and automated techniques. Int. J. Hum. Comput. Stud. 59, 213–225 10.1016/S1071-5819(03)00050-8

[B6] Camurri A. Mazzarino B. Ricchetti M. Timmers R. Volpe G. (2004). “Multimodal analysis of expressive gesture in music and dance performances,” in Gesture-Based Communication in Human-Computer Interaction. Lecture Notes in Computer Science, 2915, eds Camurri A. Volpe G. (Berlin: Springer), 20–39

[B7] Chen J. L. Penhune V. B. Zatorre R. J. (2009). The role of auditory and premotor cortex in sensorimotor transformations. Ann. N. Y. Acad. Sci. 1169, 15–34 10.1111/j.1749-6632.2009.04556.x 19673752

[B8] Collyer C. E. Broadbent H. A. Church R. M. (1992). Categorical time production: evidence for discrete timing in motor control. Percept. Psychophys. 51, 134–144 10.3758/BF03212238 1549432

[B9] Cross I. (2001). Music, cognition, culture, and evolution. Ann. N. Y. Acad. Sci. 930, 28–42 10.1111/j.1749-6632.2001.tb05723.x 11458835

[B10] Desmond J. C. (1993). Embodying difference: issues in dance and cultural studies. Cult. Crit. 26, 33–63 10.2307/1354455

[B11] Eerola T. Luck G. Toiviainen P. (2006). “An investigation of pre-schoolers’ corporeal synchronization with music,” in Proceedings of the 9th International Conference on Music Perception and Cognition, eds Baroni M. Addessi A. R. Caterina R. Costa M. (Bologna: University of Bologna), 472–476

[B12] Fraisse P. (1982). “Rhythm and tempo,” in The Psychology of Music, ed. Deutsch D. (New York, NY: Academic Press), 149–180

[B13] Godøy R. I. Haga E. Jensenius A. R. (2006). “Playing ‘Air instruments’: mimicry of sound-producing gestures by novices and experts,” in Gesture in Human-Computer Interaction and Simulation, Lecture Notes in Computer Science, 3881, eds Gibet S. Courty N. Kamp J.-F. (Berlin: Springer), 256–267

[B14] Grahn J. A. Brett M. (2007). Rhythm and beat perception in motor areas of the brain. J. Cogn. Neurosci. 19, 893–906 10.1162/jocn.2007.19.5.893 17488212

[B15] Grahn J. A. Rowe J. B. (2009). Feeling the beat: premotor and striatal interactions in musicians and nonmusicians during beat perception. J. Neurosci. 29, 7540–7548 10.1523/JNEUROSCI.2018-08.2009 19515922 PMC2702750

[B16] Hadar U. (1989). Two types of gesture and their role in speech production. J. Lang. Soc. Psychol. 8, 221–228 10.1177/0261927X8983004

[B17] Janata P. Tomic S. T. Haberman J. M. (2011). Sensorimotor coupling in music and the psychology of the groove. J. Exp. Psychol. Gen. 141, 54–75 10.1037/a0024208 21767048

[B18] Jensenius A. R. (2006). “Using motiongrams in the study of musical gestures,” in Proceedings of the International Computer Music Conference (New Orleans, LA: Tulane University), 499–502

[B19] Keller P. Rieger M. (2009). Special issue – musical movement and synchronization. Music Percept. 26, 397–400 10.1525/mp.2009.26.3.289

[B20] Lakoff G. Johnson M. (1980). Metaphors We Live By. Chicago: University of Chicago Press

[B21] Lakoff G. Johnson M. (1999). Philosophy in the Flesh: The Embodied Mind and Its Challenge to Western Thought. New York, NY: Basic Books

[B22] Lartillot O. Eerola T. Toiviainen P. Fornari J. (2008). “Multi-feature modeling of pulse clarity: design, validation, and optimization,” in Proceedings of the 9th International Conference on Music Information Retrieval, eds Bello J. P. Chew E. Turnbull D. (Philadelphia, PA: Drexel University), 521–526

[B23] Lartillot O. Toiviainen P. (2007). “A matlab toolbox for musical feature extraction from audio,” in Proceedings of the 10th International Conference on Digital Audio Effects (Bordeaux: University of Bordeaux), 1–8

[B24] Leman M. (2007). Embodied Music Cognition and Mediation Technology. Cambridge, MA: MIT Press

[B25] Leman M. Godøy R. I. (2010). “Why Study Musical Gesture?” in Musical Gestures. Sound, Movement, and Meaning, eds Godøy R. I. Leman M. (New York, NY: Routledge), 3–11

[B26] Leman M. Naveda L. (2010). Basic gestures as spatiotemporal reference frames for repetitive dance/music patterns in Samba and Charleston. Music Percept. 28, 71–92 10.1525/mp.2010.28.1.71

[B27] Lesaffre M. De Voogdt L. Leman M. De Baets B. De Meyer H. Martens J.-P. (2008). How potential users of music search and retrieval systems describe the semantic quality of music. J. Am. Soc. Inf. Sci. Technol. 59, 695–707 10.1002/asi.20731

[B28] Luck G. Saarikallio S. Burger B. Thompson M. R. Toiviainen P. (2010). Effects of the Big Five and musical genre on music-induced movement. J. Res. Pers. 44, 714–720 10.1016/j.jrp.2010.10.001

[B29] MacDougall H. G. Moore S. T. (2005). Marching to the beat of the same drummer: the spontaneous tempo of human locomotion. J. Appl. Physiol. 99, 1164–1173 10.1152/japplphysiol.00138.2005 15890757

[B30] Madison G. Gouyon F. Ullén F. Hörnström K. (2011). Modeling the tendency for music to induce movement in humans: first correlations with low-level audio descriptors across music genres. J. Exp. Psychol. Hum. Percept. Perform. 37, 1578–1594 10.1037/a0024323 21728462

[B31] Moelants D. (2002). “Preferred tempo reconsidered,” in Proceedings of the 7th International Conference on Music Perception and Cognition, eds Stevens C. Burnham D. McPherson G. Schubert E. Renwick J. (Adelaide: Causal Productions), 580–583

[B32] Murray M. P. Drought A. B. Kory R. C. (1964). Walking patterns of normal men. J. Bone Joint Surg. 46, 335–360 14129683

[B33] Naveda L. Leman M. (2010). The spatiotemporal representation of dance and music gestures using Topological Gesture Analysis (TGA). Music Percept. 28, 93–112 10.1525/mp.2010.28.1.93

[B34] Nettl B. (2000). “An ethnomusicologist contemplates universals in musical sound and musical culture,” in The Origins of Music, eds Wallin N. L. Merker B. Brown S. (Cambridge, MA: MIT Press), 463–472

[B35] Pampalk E. Rauber A. Merkl D. (2002). “Content-based organization and visualization of music archives,” in Proceedings of the 10th ACM International Conference on Multimedia, Juan-les-Pins (New York, NY: ACM Press), 570–579

[B36] Parncutt R. (1994). A perceptual model of pulse salience and metrical accent in musical rhythms. Music Percept. 11, 409–464 10.2307/40285633

[B37] Phillips-Silver J. Trainor L. J. (2008). Vestibular influence on auditory metrical interpretation. Brain Cogn. 67, 94–102 10.1016/j.bandc.2007.11.007 18234407

[B38] Repp B. H. (2005). Sensorimotor synchronization: a review of the tapping literature. Psychon. Bull. Rev. 12, 969–992 10.3758/BF03206433 16615317

[B39] Savitzky A. Golay M. J. E. (1964). Smoothing and differentiation of data by simplified least squares procedures. Anal. Chem. 36, 1627–1639 10.1021/ac60214a047

[B40] Shrout P. E. Fleiss J. L. (1979). Intraclass correlations: uses in assessing rater reliability. Psychol. Bull. 86, 420–428 10.1037/0033-2909.86.2.420 18839484

[B41] Stevens C. J. Schubert E. Wang S. Kroos C. Halovic S. (2009). Moving with and without music: scaling and lapsing in time in the performance of contemporary dance. Music Percept. 26, 451–464 10.1525/mp.2009.26.5.451

[B42] Styns F. van Noorden L. Moelants D. Leman M. (2007). Walking on music. Hum. Mov. Sci. 26, 769–785 10.1016/j.humov.2007.07.007 17910985

[B43] Toiviainen P. Burger B. (2011). MoCap Toolbox Manual. Jyväskylä: University of Jyväskylä

[B44] Toiviainen P. Luck G. Thompson M. (2010). Embodied meter: hierarchical eigenmodes in music-induced movement. Music Percept. 28, 59–70 10.1525/mp.2010.28.1.1

[B45] Trainor L. J. Gao X. Lei J.-J. Lehtovaara K. Harris L. R. (2009). The primal role of the vestibular system in determining musical rhythm. Cortex 45, 35–43 10.1016/j.cortex.2007.10.014 19054504

[B46] Van Dyck E. Moelants D. Demey M. Coussement P. Deweppe A. Leman M. (2010). “The impact of the bass drum on body movement in spontaneous dance,” in Proceedings of the 11th International Conference in Music Perception and Cognition, eds Demorest S. M. Morrison S. J. Campbell P. S. (Seattle, WA: University of Washington), 429–434

[B47] Varela F. J. Thompson E. Rosch E. (1991). The Embodied Mind: Cognitive Science and Human Experience. Cambridge, MA: MIT Press

[B48] Zentner M. Eerola T. (2010). Rhythmic engagement with music in infancy. Proc. Natl. Acad. Sci. U.S.A. 107, 5768–5773 10.1073/pnas.1000121107 20231438 PMC2851927

